# Self-Reported Knowledge and Awareness of Musician-Related Disorders Among Music Educators and Pre-Service Music Teachers in Higher Education Institutions in Türkiye: An Occupational Health Literacy Perspective

**DOI:** 10.3390/healthcare14172811

**Published:** 2026-09-02

**Authors:** Rıza Akyürek, Ayşe Şevval Dinçer

**Affiliations:** 1 Department of Fine Arts Education, Faculty of Education, Muğla Sıtkı Koçman University, Muğla 48100, Türkiye; 2 Graduate School of Educational Sciences, Muğla Sıtkı Koçman University, Muğla 48100, Türkiye; sevvaldincer1@gmail.com

**Keywords:** occupational health literacy, musician health, musician-related disorders, performance-related musculoskeletal disorders, music teacher education, occupational well-being

## Abstract

Background/Objectives: Musicians face occupational health risks from repetitive movement, sustained posture, and intensive practice. This study examined self-reported knowledge of musician-related disorders, physical complaints, and selected health practices among music educators and pre-service music teachers, using occupational health literacy (OHL) as an interpretive framework. Methods: A cross-sectional survey included 432 participants from public higher education institutions in Türkiye (83 educators; 349 pre-service teachers). The primary outcome was a common 15-item self-reported disorder knowledge score (range 15–75). Psychometric and nonparametric analyses included effect sizes, confidence intervals, and multiplicity correction. Results: Instrument-related physical problems were reported by 49.4% of educators and 53.0% of pre-service teachers. After false discovery rate correction, hand/wrist complaints were more frequent among pre-service teachers. The score showed a one-factor structure and excellent internal consistency. Educators (*n* = 79; M = 38.73, SD = 14.71) scored higher than pre-service teachers (*n* = 341; M = 26.81, SD = 12.35; U = 20,062.0, *p* < 0.001; rank biserial r = 0.489, 95% CI 0.364–0.602). Conclusions: Knowledge was uneven, especially for specialized conditions. Targeted musician-health education, communication, and referral pathways are warranted. The score is not a comprehensive OHL measure or a test of clinical competence, and causal effects on preventive behavior or occupational well-being cannot be inferred.

## 1. Introduction

Musical performance combines repetitive motor activity, sustained or asymmetric postures, continuous neuromuscular coordination, and prolonged periods of concentrated practice. These demands support artistic performance but also expose musicians to physical and psychological occupational health risks [1,2,3]. Performance-related musculoskeletal disorders (PRMDs) comprise pain, weakness, numbness, fatigue, loss of motor control, and other symptoms that interfere with playing. The neck, shoulders, back, forearms, wrists, hands, and fingers are frequently affected [4,5,6], while performance anxiety, occupational stress, and burnout may interact with pain and functional limitations [7,8].

The clinical and educational relevance of musician health extends beyond common pain complaints. Musicians may experience tendinitis, nerve entrapment syndromes, thoracic outlet syndrome, focal dystonia, task-specific tremor, temporomandibular disorders, postural abnormalities, and other instrument- or task-related conditions [9,10,11]. Performing arts medicine therefore emphasizes prevention, early symptom recognition, workload management, ergonomic support, conditioning, and timely referral rather than treatment alone [12,13,14,15]. Higher education institutions are particularly important because practice routines, postural habits, and attitudes toward injury are formed during professional preparation.

Health literacy describes the ability to access, understand, appraise, communicate, and use health information [15,16]. Occupational health literacy (OHL) extends these competencies to work-related risks and health decisions. It is commonly discussed through functional, interactive, and critical dimensions [17,18,19]. In music education, functional OHL may involve basic knowledge of performance-related disorders and warning signs; interactive OHL may involve communication and help seeking; and critical OHL may involve evaluating information and selecting appropriate preventive or referral actions. However, knowledge of disorder names or symptoms alone does not constitute comprehensive OHL, clinical knowledge, or diagnostic competence.

Music educators influence students’ practice schedules, posture, preparation strategies, symptom communication, and attitudes toward professional support. Studies of higher education musicians have identified gaps in health awareness, access to structured health education, and consistent preventive behavior [20,21,22,23,24]. These gaps may affect sustainable participation in music education and, potentially, occupational well-being. Research has nevertheless focused more often on symptom prevalence than on the coexistence of physical complaints, disorder-specific knowledge, communication, and guidance within music teacher education.

The present study addresses this gap using a multi-institutional sample of music educators and pre-service music teachers in Türkiye. OHL is used only as an interpretive perspective; it is not treated as a directly measured multidimensional outcome. The primary measured outcome is a common 15-item self-reported musician-related disorder knowledge score. This study also describes physical complaints and selected musician health practices. The following hypotheses were evaluated:

**H1.**  *Knowledge scores differ according to gender within the music educator and pre-service music teacher groups.*

**H2.**  *Participants reporting regular physical exercise have higher knowledge scores than participants not reporting regular exercise within each group.*

**H3.**  *Music educators have higher knowledge scores than pre-service music teachers on the common 15-item score.*

**H4.**  *Among music educators, knowledge scores differ according to educational status and are associated with age.*

**H5.**  *Among pre-service music teachers, knowledge scores differ according to year of study and years of instrumental experience.*

Item-level disorder comparisons and body map comparisons between groups were prespecified as exploratory analyses and evaluated with false discovery rate correction. All hypotheses concern associations or group differences in cross-sectional data and do not imply causality.

## 2. Materials and Methods

### 2.1. Research Design

This quantitative cross-sectional survey described self-reported knowledge of musician-related disorders, physical complaints, and selected musician health experiences and examined group differences and associations. Data were collected during the fall and spring semesters of the 2023–2024 and 2024–2025 academic years through face-to-face and online administration. The same role-appropriate instruments, eligibility criteria, item wording, instructions, response options, and scoring procedures were used throughout both academic years; only the delivery mode differed according to participant access. OHL provided the interpretive framework, but no separate instrument measuring comprehensive functional, interactive, and critical OHL was administered.

### 2.2. Participants

The target population comprised academic staff and undergraduate students in music education divisions within departments of fine arts education at public universities in Türkiye. In this article, “music educators” refers to academic staff teaching individual instrument, voice, and/or other professional music-related courses; the term is not restricted to individual instrument teachers. Pre-service music teachers were first- through fourth-year undergraduate students enrolled in the participating programs.

A total of 550 individuals were invited, 450 questionnaires were returned (initial response rate 81.8%), and 18 invalid questionnaires were excluded during data screening. The final sample comprised 432 participants (78.5% of invitees and 96.0% of returned questionnaires): 83 music educators and 349 pre-service music teachers. Among educators, 38 were women (45.8%) and 45 were men (54.2%); mean age was 45.49 years (SD = 10.18; range 19–65). Among pre-service teachers, gender was reported by 348 participants (215 women, 61.8%; 133 men, 38.2%) and was missing for one participant; age was available for 339 participants (M = 22.54, SD = 4.80; range 18–48). Participation was voluntary and institution-level sample sizes were not proportional.

Educators were eligible if they were employed in a participating music education division during the study period and consented voluntarily. Pre-service teachers were eligible if they were enrolled in a participating program during the study period and consented voluntarily. Participants who left the relevant position or program during data collection, and questionnaires deemed invalid during screening, were excluded. Detailed participant characteristics are provided in Supplementary Table S1A,B. Institution-level counts are summarized in coded form because several educator cells were small and potentially identifiable.

### 2.3. Instruments and Scoring

Data were collected with group-specific questionnaires based on the musician disorder knowledge level forms developed in Turkish by Satıcı et al. [25]. The questionnaires were administered in Turkish; therefore, no translation or cross-cultural language adaptation procedure was required. The versions differed in their demographic and role-specific questions but contained an identical 15-item disorder knowledge block.

The educator questionnaire contained 17 non-Likert fields, 25 five-point Likert-type items, and an optional comment field. The pre-service teacher questionnaire contained 14 non-Likert fields, 25 five-point Likert-type items, and an optional comment field. In both questionnaires, the first 10 Likert-type items addressed heterogeneous contextual experiences and practices, including physical complaints, rest, professional consultation or health information, communication, posture guidance, and warm-up/relaxation guidance. Because these items did not represent a single latent construct, they were interpreted individually and were not combined into a scale score.

The remaining 15 items asked participants to rate their knowledge of overuse syndrome, tendinitis, focal dystonia, carpal tunnel syndrome, thoracic outlet syndrome, cubital tunnel syndrome, trigger finger, entrapment neuropathies, disc herniation, temporomandibular disorder, dental alignment problems, task-specific tremor, Fiddler’s neck, scoliosis, and kyphosis. Responses ranged from 1 (“none”) to 5 (“completely”); no item was reverse-scored. Complete responses were summed to produce the self-reported musician-related disorder knowledge score (range 15–75), with higher scores indicating greater perceived disorder-specific knowledge. The score is not an objective knowledge test, an assessment of diagnostic competence, or a comprehensive OHL measure.

The body map comprised 22 anatomical regions. Participants could report multiple regions; each region was coded separately as 0 (“not reported”) or 1 (“reported”), and percentages were therefore not mutually exclusive. Printed and online questionnaires used the same wording and response format. The item map, scoring rules, and representative items are provided in Supplementary Table S2.

### 2.4. Validity and Reliability

The original instrument development procedures are reported by Satıcı et al. [25]. In the present study, psychometric evaluation was restricted to the 15 items that were identical across both questionnaires. Here, reliability refers to the psychometric internal consistency of item responses, not the operational reliability of a device or system. Complete 15-item responses were available for 79 educators and 341 pre-service teachers.

The educator score showed excellent internal consistency (Cronbach’s α = 0.962, 95% CI 0.944–0.974; McDonald’s ω = 0.963), as did the pre-service teacher score (α = 0.946, 95% CI 0.933–0.956; ω = 0.949). Corrected item–total correlations ranged from 0.605 to 0.876 among educators and from 0.601 to 0.818 among pre-service teachers. Sampling adequacy was high (Kaiser–Meyer–Olkin = 0.913 and 0.939), and Bartlett’s tests of sphericity were significant for educators (χ^2^(105) = 1201.55, *p* < 0.001) and pre-service teachers (χ^2^(105) = 3929.77, *p* < 0.001).

Parallel analysis supported retention of one factor in each group. Separate one-factor exploratory factor analyses of the item correlation matrices using maximum likelihood extraction yielded standardized loadings of 0.594–0.889 among educators and 0.574–0.842 among pre-service teachers. Detailed loadings, item total correlations, and α-if-item-deleted values are provided in Supplementary Table S3. These findings support internal consistency and a one-factor observed score interpretation in the present samples; they do not validate the questionnaires as comprehensive multidimensional OHL instruments or establish measurement invariance across groups.

### 2.5. Data Analysis

Analyses were conducted using Python 3.13.5 with pandas 2.2.3, NumPy 2.3.5, SciPy 1.17.0, and statsmodels 0.14.6. Likert labels were standardized and coded from 1 to 5; entries outside the predefined categories were treated as missing. No missing values were imputed. Item-level statistics and body map frequencies used all valid responses for the relevant variable. Calculation of the composite score required complete responses to all 15 items. Analyses involving grouping variables additionally excluded cases missing that variable, and valid n is reported for every analysis.

Categorical variables were summarized as frequencies and percentages. Likert items and total scores were summarized with means and standard deviations and, where relevant, medians and interquartile ranges. Distributional shape was assessed with histograms, quantile–quantile plots, and Shapiro–Wilk tests. Because composite scores departed from normality and were derived from ordinal responses, two-sided Mann–Whitney U tests were used for two-group comparisons, Kruskal–Wallis tests for comparisons involving more than two groups, and Spearman’s rank correlation for the association between educator age and score. Significant Kruskal–Wallis tests were followed by pairwise Mann–Whitney tests with Holm adjustment.

The primary educator–pre-service teacher comparison used the identical 15-item block. The 15 item-level comparisons were evaluated with Mann–Whitney U tests and Benjamini–Hochberg false discovery rate (FDR) adjustment. The 22 binary body map comparisons used Fisher’s exact tests with Benjamini–Hochberg adjustment. Mann–Whitney effects are reported as rank biserial correlations with percentile bootstrap 95% confidence intervals; Kruskal–Wallis effects as ε^2^; correlations as Spearman’s ρ; and body map effects as odds ratios (ORs) with 95% confidence intervals. Prespecified hypotheses used α = 0.05; exploratory families used q < 0.05. A Holm sensitivity adjustment across the eight secondary within-group tests was also examined; because none survived that family-wise adjustment, secondary findings with nominal *p* < 0.05 were interpreted as exploratory rather than confirmatory.

No formal a priori sample-size calculation was recorded because recruitment was conducted across participating institutions during the study period. A sensitivity analysis based on the complete score samples (79 educators and 341 pre-service teachers), two-sided α = 0.05, and 80% power indicated that the primary comparison could detect an approximate standardized mean difference of d = 0.35.

During revision, ChatGPT 5.6 (OpenAI) was used to assist with drafting statistical code. The tool was not used to generate or alter study data; all analyses and reported outputs were executed and verified by the authors against the study data.

### 2.6. Ethical Considerations

This study was conducted in accordance with the Declaration of Helsinki and approved by the Social and Human Sciences Research Ethics Committee-1 of Muğla Sıtkı Koçman University on 29 September 2023 (Protocol No. 230083; Decision No. 81). Participation was voluntary. Face-to-face participants received printed information and provided written informed consent; online participants approved an electronic consent statement before accessing the questionnaire. Participants were informed of the purpose and scope of the study, confidentiality, and their right to withdraw without penalty. Data were anonymized before analysis.

## 3. Results

### 3.1. Health Characteristics and Body Map Findings

Among educators, 44 (53.0%) reported regular physical exercise, 41 (49.4%) reported an instrument-related physical problem, and 17 (20.5%) reported a condition diagnosed by a physician or physiotherapist; among pre-service teachers, the corresponding frequencies were 97 (27.8%), 185 (53.0%), and 29 (8.3%), respectively (Table 1).

The neck was the most frequently reported region in both groups (33.7% of educators and 39.0% of pre-service teachers). After FDR correction across 22 regions, only right hand/wrist (q < 0.001) and left hand/wrist (q = 0.003) differences remained significant, with both complaints reported more frequently by pre-service teachers. The unadjusted right upper arm difference (*p* = 0.008) did not remain significant after correction (q = 0.056) (Table 2). Full results for all 22 regions are provided in Supplementary Table S4.

### 3.2. Disorder-Specific Self-Reported Knowledge

Knowledge ratings varied across disorders (Table 3). Educators reported the highest means for overuse syndrome (M = 3.07, SD = 1.15), scoliosis (M = 3.01, SD = 1.23), and kyphosis (M = 2.89, SD = 1.27), and the lowest means for dental alignment problems (M = 2.04, SD = 1.21) and task-specific tremor (M = 2.04, SD = 1.09). Pre-service teachers reported the highest means for scoliosis (M = 2.33, SD = 1.32), kyphosis (M = 2.13, SD = 1.29), and overuse syndrome (M = 2.09, SD = 1.12), and the lowest for dental alignment problems (M = 1.50, SD = 0.88) and task-specific tremor (M = 1.55, SD = 0.91).

Educator ratings were higher for all 15 shared items, and every item-level difference remained significant after FDR correction (all q < 0.001). Rank biserial effects ranged from 0.251 to 0.507; the largest difference concerned tendinitis and the smallest concerned dental alignment problems.

### 3.3. Composite Knowledge Score and Participant Role Comparison

Complete scores were available for 79 educators and 341 pre-service teachers. Educators had a mean score of 38.73 (SD = 14.71; median = 38, IQR = 28–49), and pre-service teachers had a mean of 26.81 (SD = 12.35; median = 23, IQR = 17–32) (Table 4). The groups differed significantly (U = 20,062.0, *p* < 0.001), with a moderate-to-large effect favoring educators (rank biserial r = 0.489, 95% CI 0.364–0.602). H3 was therefore supported for the common observed score.

### 3.4. Within-Group Differences in Knowledge Scores

Among educators, the gender difference was not statistically significant (U = 956.5, *p* = 0.066). Regular exercise (U = 977.5, *p* = 0.049; rank biserial r = 0.258) and age (ρ = −0.257, *p* = 0.022) met the nominal threshold, whereas educational status differences did not (H(3) = 7.649, *p* = 0.054; ε^2^ = 0.062) (Table 5). Among pre-service teachers, gender (U = 14,780.0, *p* = 0.232) and instrumental experience (H(3) = 4.503, *p* = 0.212; ε^2^ = 0.004) were not significant. Regular exercise (U = 13,398.5, *p* = 0.027; rank biserial r = 0.154) and year of study (H(3) = 9.860, *p* = 0.020; ε^2^ = 0.020) met the nominal threshold; the only Holm-adjusted pairwise year comparison was fourth year versus second year (adjusted *p* = 0.023; rank biserial r = 0.242) (Table 6).

In the sensitivity analysis adjusting across all eight secondary within-group tests, none remained significant (Holm-adjusted *p* values 0.079–0.424). Accordingly, H1 was not supported; the findings related to exercise, age, and year of study are treated as tentative exploratory signals rather than confirmatory evidence. H4 and H5 received only partial nominal support.

### 3.5. Health Information, Guidance, and Related Practices

The contextual items showed mixed musician health support and communication. Among educators, 67.5% reported consulting a health specialist never or very little, whereas 62.7% reported informing students about potential physical problems largely or completely, 66.3% reported reminders about actions before, during, and after practice, 95.2% reported posture guidance, and 72.3% reported information about physical structures at risk. Among pre-service teachers, 67.0% reported receiving no or very little information about their physical complaints, 66.5% reported sharing problems with their instrument educator never or very little, and 39.4% reported no guidance about a specialist-designed warm-up or relaxation program. Posture guidance was more frequently reported. These heterogeneous items were interpreted individually and were not included in the composite score (Table 7).

## 4. Discussion

### 4.1. Physical Complaints and PRMD Patterns

Approximately half of educators and pre-service teachers reported an instrument-related physical problem, whereas professionally diagnosed conditions were less frequent. These results distinguish perceived complaints from medically confirmed conditions and should not be interpreted as clinical prevalence estimates. The concentration of complaints in the neck, shoulders, hands, wrists, and forearms is consistent with international research on PRMDs [1,2,3,4,5,6]. Repetition, asymmetric posture, instrument-specific biomechanics, workload, and insufficient recovery may contribute to these patterns, but the present study cannot identify causes. Comparable patterns have been reported among higher education music students, professional instrumentalists, and younger instrumentalists, with practice exposure, posture, instrument demands, and psychosocial factors repeatedly identified as relevant correlates of musculoskeletal complaints [26,27,28,29,30,31,32].

After multiplicity correction, only right and left hand/wrist complaints were significantly more frequent among pre-service teachers. This finding narrows the interpretation that would have followed from unadjusted comparisons and suggests that hand/wrist health may deserve particular attention during teacher preparation. Symptom recognition, practice load planning, recovery, and referral information could be introduced before complaints become persistent or performance-limiting [20,21,33].

### 4.2. Disorder-Specific Knowledge and the OHL Perspective

Knowledge was uneven. Both groups were relatively more familiar with widely recognized musculoskeletal or postural conditions, especially overuse syndrome, scoliosis, and kyphosis, and less familiar with specialized neurological, entrapment, temporomandibular, and task-specific conditions. General musculoskeletal terminology may be encountered in everyday health information or personal experience, whereas specialist performing arts conditions may require explicit instruction. This explanation is plausible but was not directly tested. The literature on musician’s dystonia further shows that specialized performance-related conditions involve complex neurological and psychological characteristics that are unlikely to be understood adequately through general health knowledge alone [34].

All item-level ratings and the total observed score were higher among educators. Nevertheless, educators also showed limited knowledge of several specialist conditions. Curricula should therefore address both common pain/posture problems and occupation-specific warning signs, while maintaining clear referral boundaries. From an OHL perspective, the score reflects a coherent content-specific knowledge component most closely related to functional literacy. It does not demonstrate critical appraisal, communication competence, clinical decision making, diagnosis, or effective preventive behavior [15,16,17,18,19].

### 4.3. Participant Role and Secondary Group Differences

The identical 15-item block permitted a direct observed score comparison, and educators scored higher with a moderate-to-large effect. Professional exposure, teaching responsibilities, or contact with musician health information may contribute, but the cross-sectional design cannot determine which experiences produced the difference. Measurement invariance was not tested; the finding should therefore be interpreted as an observed score comparison rather than a latent mean comparison.

Gender differences were not supported. Exercise, educator age, and pre-service teachers’ year of study produced small nominal associations, but none of the eight secondary tests survived the global Holm sensitivity adjustment. These findings should consequently be regarded as hypothesis-generating. They do not show that exercise increases knowledge or that age reduces it. The absence of robust differences according to education level or instrumental experience suggests that knowledge may not increase consistently through informal exposure alone.

### 4.4. Health Communication and Curriculum Implications

Educators frequently reported providing posture and risk structure guidance, yet many reported little consultation with health professionals. Pre-service teachers reported limited information about complaints, limited warm-up/relaxation guidance, and infrequent communication with instrument educators. Because educators and students were not matched as teacher–student pairs, these reports are not direct contradictions; they may reflect differences in recall, timing, specificity, or interpretation. Qualitative and educational case studies likewise indicate that musicians may normalize symptoms, delay help seeking, or experience uncertainty about where to obtain musician-specific support, while structured university-based health education can provide a feasible route for improving awareness [35,36].

Curriculum development should extend beyond general posture reminders. Structured content may include common and specialized disorders, early warning signs, ergonomic principles, workload and recovery planning, communication about symptoms, and criteria for professional referral. Professional development and interdisciplinary workshops may strengthen consistency, but their effects on behavior, injury, or health outcomes require intervention research [20,21,22,23,24]. Recent systematic and qualitative evidence also supports combining education with appropriately designed physical conditioning, prevention, and referral components, while emphasizing that intervention content and long-term effectiveness remain heterogeneous [37,38].

### 4.5. Theoretical Contribution and Occupational Well-Being

This study’s theoretical contribution is deliberately bounded: it applies OHL as an interpretive perspective while separating that framework from the measured score. Strong internal consistency and a one-factor solution support interpretation of the 15 items as self-reported disorder-specific knowledge in these samples. They do not establish comprehensive functional, interactive, and critical OHL. Contextual findings on consultation, communication, and access to guidance may be relevant to interactive health literacy, but they were analyzed as individual indicators. More broadly, health literacy measurement is context-sensitive across populations and occupational domains [39,40], and the musicians’ health literacy questionnaire provides a musician-specific model that assesses access, understanding, appraisal, and application of performance health information [41].

Persistent complaints, limited specialist knowledge, and inconsistent access to guidance may have implications for perceived health, functional capacity, confidence, career sustainability, and workers’ well-being among educators and future educators [24,42]. Conversely, clearer information, earlier recognition, and appropriate referral may support safer participation in musical work and training. Well-being was not directly measured, and this link is an occupational health implication rather than a demonstrated outcome. Recent occupational health research has also linked knowledge- and skill-based OHL with work ability, while highlighting the contribution of organizational and leadership conditions; this reinforces the need to interpret musician health as both an individual and institutional responsibility [43].

### 4.6. Strengths, Limitations, and Future Research

Strengths include the multi-institutional sample, reanalysis of the original raw data, use of an identical 15-item block for the primary comparison, reporting of effect sizes and confidence intervals, psychometric evaluation in both groups, and multiplicity correction for item-level and body map families.

Several limitations require caution. The cross-sectional design precludes temporal or causal inference. All principal variables were self-reported and may be affected by recall, subjective interpretation, and social desirability; complaints were not clinically verified. This study did not administer a comprehensive validated OHL instrument. The exploratory factor analyses were conducted in the same samples used for substantive analyses, no independent confirmatory factor analysis or measurement invariance testing was performed, and ordinal item models using polychronic correlations were not evaluated. Complete case scoring reduced the analytic samples to 79 educators and 341 pre-service teachers, and no imputation was used. Participation was voluntary and institution-level contributions were unequal, limiting generalizability. Face-to-face and online administration modes were not compared. Body map data were multiple response reports, and group comparisons did not adjust for instrument type, practice exposure, or other potential confounders. No a priori sample-size calculation was recorded. Finally, secondary within-group findings did not survive the global Holm sensitivity adjustment.

Future research should validate musician-specific OHL instruments that assess functional, interactive, and critical competencies; confirm the factor structure and measurement invariance in independent samples; and use longitudinal or intervention designs to test whether education improves communication, preventive practices, well-being, or health outcomes. Clinical assessment, ergonomic observation, and objective workload measures would strengthen future evidence. Broader samples should include conservatory students, professional performers, private institutions, and international populations.

## 5. Practical Implications

As an immediate priority, existing instrument, voice, pedagogy, and professional development courses can incorporate concise modules on ergonomics, early warning symptoms, safe practice scheduling, rest and recovery, and referral indications. Specialist conditions with low ratings—such as focal dystonia, thoracic outlet syndrome, cubital tunnel syndrome, trigger finger, temporomandibular disorder, task-specific tremor, and Fiddler’s neck—deserve explicit attention.

As a medium-term priority, departments can provide structured educator development and interdisciplinary workshops involving physiotherapists, occupational therapists, physicians, psychologists, audiologists, and occupational health professionals. Simple referral pathways can clarify where students and educators should seek assistance for persistent or performance-limiting symptoms.

As a longer-term institutional goal, universities can develop coordinated musician health systems integrating prevention education, consultation, and referral. These recommendations respond to observed gaps; their effectiveness must be evaluated rather than assumed.

## 6. Conclusions

This multi-institutional cross-sectional study examined physical complaints, contextual musician health practices, and a common 15-item self-reported disorder knowledge score among 83 music educators and 349 pre-service music teachers in Türkiye. Approximately half of both groups reported instrument-related physical problems. After FDR correction, right and left hand/wrist complaints were more frequent among pre-service teachers.

Educators scored higher than pre-service teachers on the common knowledge score, with a moderate-to-large observed effect, but both groups showed gaps for specialist conditions. Gender differences were not supported, and secondary associations were tentative after family-wise sensitivity analysis. The findings support targeted education, communication, warm-up and recovery guidance, and referral pathways. They do not establish clinical competence, comprehensive OHL, causal effects on preventive behavior, or improvements in occupational well-being.

## Figures and Tables

**Table 1:** Regular exercise, instrument-related physical problems, and professionally diagnosed conditions.

Indicator	Music Educators, n (%)	Pre-Service Teachers, n (%)
Regular physical exercise	44 (53.0)	97 (27.8)
Instrument-related physical problem	41 (49.4)	185 (53.0)
Professionally diagnosed condition	17 (20.5)	29 (8.3)

Note: Percentages are based on 83 music educators and 349 pre-service music teachers.

**Table 2:** Principal body map complaint regions and educator/pre-service teacher comparisons.

Anatomical Region	Educators, n (%)	Pre-Service, n (%)	OR	95% CI	Fisher *p*	BH-FDR q
Neck	28 (33.7)	136 (39.0)	0.80	0.48–1.32	0.450	0.874
Right shoulder/posterior shoulder	21 (25.3)	93 (26.6)	0.93	0.54–1.61	0.890	1.000
Left shoulder/posterior shoulder	23 (27.7)	105 (30.1)	0.89	0.52–1.52	0.789	1.000
Right upper arm	3 (3.6)	48 (13.8)	0.24	0.07–0.78	0.008	0.056
Left upper arm	7 (8.4)	39 (11.2)	0.73	0.32–1.70	0.556	0.874
Right forearm	9 (10.8)	52 (14.9)	0.70	0.33–1.47	0.386	0.874
Left forearm	12 (14.5)	62 (17.8)	0.78	0.40–1.53	0.521	0.874
Right hand/wrist	11 (13.3)	128 (36.7)	0.26	0.14–0.52	<0.001	<0.001
Left hand/wrist	15 (18.1)	135 (38.7)	0.35	0.19–0.64	<0.001	0.003
Right abdomen/lower back	4 (4.8)	37 (10.6)	0.43	0.15–1.23	0.143	0.630
Left abdomen/lower back	4 (4.8)	36 (10.3)	0.44	0.15–1.27	0.143	0.630

Note: ORs express the odds among music educators relative to pre-service music teachers; values below 1 indicate higher reporting among pre-service teachers. Multiple regions could be selected. All 22 comparisons were included in the FDR adjustment.

**Table 3:** Disorder-specific knowledge ratings and FDR-adjusted group comparisons.

Disorder	Educators: n; M ± SD	Pre-Service: n; M ± SD	U	*p*	BH-FDR q	r_rb
Overuse syndrome	83; 3.07 ± 1.15	349; 2.09 ± 1.12	21,082.5	<0.001	<0.001	0.456
Tendinitis	83; 2.83 ± 1.22	348; 1.73 ± 1.06	21,771.0	<0.001	<0.001	0.507
Focal dystonia	82; 2.43 ± 1.22	348; 1.76 ± 1.11	19,008.0	<0.001	<0.001	0.332
Carpal tunnel syndrome	82; 2.84 ± 1.21	348; 1.95 ± 1.21	20,065.5	<0.001	<0.001	0.406
Thoracic outlet syndrome	81; 2.47 ± 1.16	346; 1.69 ± 1.03	19,509.5	<0.001	<0.001	0.392
Cubital tunnel syndrome	82; 2.38 ± 1.19	346; 1.66 ± 1.08	19,409.5	<0.001	<0.001	0.368
Trigger finger	81; 2.31 ± 1.27	348; 1.63 ± 1.01	18,611.5	<0.001	<0.001	0.321
Entrapment neuropathies	82; 2.54 ± 1.25	348; 1.74 ± 1.10	19,630.0	<0.001	<0.001	0.376
Disc herniation	82; 2.82 ± 1.25	348; 1.91 ± 1.13	20,127.0	<0.001	<0.001	0.411
Temporomandibular disorder	82; 2.23 ± 1.25	347; 1.62 ± 0.98	18,412.0	<0.001	<0.001	0.294
Dental alignment problems	82; 2.04 ± 1.21	346; 1.50 ± 0.88	17,741.0	<0.001	<0.001	0.251
Task-specific tremor	81; 2.04 ± 1.09	347; 1.55 ± 0.91	17,833.0	<0.001	<0.001	0.269
Fiddler’s neck	82; 2.44 ± 1.31	348; 1.76 ± 1.14	18,814.0	<0.001	<0.001	0.319
Scoliosis	82; 3.01 ± 1.23	348; 2.33 ± 1.32	18,590.5	<0.001	<0.001	0.303
Kyphosis	82; 2.89 ± 1.27	348; 2.13 ± 1.29	19,030.0	<0.001	<0.001	0.334

Note: Items were rated from 1 (none) to 5 (completely). Positive rank biserial correlations indicate higher ratings among educators. Item-level analyses used all valid responses for each item.

**Table 4:** Summary of the 15-item self-reported musician-related disorder knowledge score.

Participant Group	n	M ± SD	Median (IQR)	95% CI for Mean
Music educators	79	38.73 ± 14.71	38 (28–49)	35.44–42.03
Pre-service music teachers	341	26.81 ± 12.35	23 (17–32)	25.49–28.12

Note: Possible scores ranged from 15 to 75; higher scores indicate greater self-reported disorder-specific knowledge.

**Table 5:** Knowledge score comparisons and associations among music educators.

Variable	Category	n	M ± SD	Median (IQR)	Test	*p*	Effect Size (95% CI)
Gender	Female	35	42.57 ± 15.88	40 (32–50)	U = 956.5	0.066	rank biserial r = 0.242 (−0.007 to 0.483)
	Male	44	35.68 ± 13.11	33.5 (27–46)			
Regular exercise	Yes	42	41.95 ± 15.78	42.5 (29.5–50)	U = 977.5	0.049	rank biserial r = 0.258 (0.008 to 0.496)
	No	37	35.08 ± 12.64	34 (27–44)			
Educational status	Bachelor’s	7	33.14 ± 9.10	34 (27.5–39)	H(3) = 7.649	0.054	ε^2^ = 0.062
	Master’s	15	33.13 ± 9.71	32 (27.5–40)			
	Doctorate	54	40.13 ± 15.93	39.5 (27.25–49.75)			
	Proficiency in art	3	54.67 ± 5.51	55 (52–57.5)			
Age	Continuous	79	-	-	ρ = −0.257	0.022	Spearman ρ = −0.257

Note: Analyses used complete 15-item scores. Confidence intervals for rank biserial correlations were obtained by percentile bootstrap. Nominal *p* values are shown; no secondary test survived the global Holm sensitivity adjustment.

**Table 6:** Knowledge score comparisons among pre-service music teachers.

Variable	Category	n	M ± SD	Median (IQR)	Test	*p*	Effect Size (95% CI)
Gender	Female	208	27.32 ± 12.59	23 (17–32)	U = 14,780.0	0.232	rank biserial r = 0.077 (−0.048 to 0.204)
	Male	132	25.97 ± 12.00	21 (16–33)			
Regular exercise	Yes	94	29.29 ± 13.30	25 (18–38.75)	U = 13,398.5	0.027	rank biserial r = 0.154 (0.021 to 0.292)
	No	247	25.87 ± 11.86	22 (17–31)			
Year of study	1st year	94	25.63 ± 11.84	21 (16–31)	H(3) = 9.860	0.020	ε^2^ = 0.020
	2nd year	103	25.23 ± 11.87	21 (16–31)			
	3rd year	56	26.68 ± 12.08	22.5 (18–32)			
	4th year	88	30.00 ± 13.17	27 (20–39.25)			
Instrumental experience	0–5 years	192	26.17 ± 12.50	22 (16–32)	H(3) = 4.503	0.212	ε^2^ = 0.004
	6–10 years	102	26.85 ± 11.72	23 (17–32.75)			
	11–15 years	37	28.38 ± 12.03	25 (20–34)			
	≥16 years	10	32.80 ± 16.26	28 (19.5–45.75)			

Note: The gender analysis included 340 participants with complete scores and non-missing gender data. The fourth-year versus second-year comparison was the only pairwise year contrast significant after within-analysis Holm adjustment; no secondary test survived the global Holm sensitivity adjustment.

**Table 7:** Selected contextual musician health items by participant group.

Group	Item	Valid n	M ± SD	Most Frequent Response	n (%)
Educators	Frequency of consulting a specialist for physical health	83	2.14 ± 1.13	None	30 (36.1)
Educators	Feedback from students about pain/numbness/loss of sensation	83	2.28 ± 0.86	Very little	31 (37.3)
Educators	Information provided about potential physical problems	83	3.75 ± 1.01	Largely	31 (37.3)
Educators	Reminders about actions before, during, and after practice	83	3.86 ± 0.94	Largely	32 (38.6)
Educators	Posture guidance	83	4.60 ± 0.62	Completely	55 (66.3)
Educators	Information about physical structures at risk	83	4.06 ± 0.93	Completely	33 (39.8)
Pre-service	Information received about physical complaints	345	2.10 ± 1.03	None	121 (35.1)
Pre-service	Sharing problems with instrument educator	349	2.16 ± 1.17	None	129 (37.0)
Pre-service	Information about potential physical problems	348	2.81 ± 1.37	Partially	84 (24.1)
Pre-service	Information on specialist-designed warm-up/relaxation	348	2.37 ± 1.39	None	137 (39.4)
Pre-service	Posture guidance	348	3.83 ± 1.25	Completely	143 (41.1)

Note: Response options ranged from 1 (none) to 5 (completely). Percentages use valid responses for each item. Contextual items were not combined into a scale.

## Data Availability

The data presented in this study are available from the corresponding author on reasonable request. The data are not publicly available because of participant privacy and ethical restrictions.

## Supplementary Materials

The following supporting information can be downloaded at: https://www.mdpi.com/article/10.3390/healthcare14172811/s1, Table S1A: Participant characteristics; Table S1B. Principal Instrument/Voice Specializations; Table S2: Questionnaire structure, scoring, and representative items; Table S3: Item-level psychometric results for the 15-item knowledge score; Table S4: Complete body map comparison results.
