# Supplementary material for: Self-Reported Knowledge and Awareness of Musician-Related Disorders Among Music Educators and Pre-Service Music Teachers in Higher Education Institutions in Türkiye: An Occupational Health Literacy Perspective
Source: Healthcare (Basel). 2026 Sep 2;14(17):2811. doi: 10.3390/healthcare14172811 (PMC13565113; doi:10.3390/healthcare14172811)
Supplement: Supplementary file 1 [file healthcare-14-02811-s001.zip › healthcare-4453983-supplementary.pdf]

Supplementary Materials

Self-Reported Knowledge and Awareness of Musician-Related Disorders Among Music Educators and Pre-Service Music Teachers in Higher Education Institutions in Türkiye: An Occupational Health Literacy Perspective

Rıza Akyürek and Ayşe Şevval Dinçer

Table S1A. Participant Characteristics

Percentages use the relevant group denominator unless a different valid n is stated. Institution-level sample sizes are summarized without names because several educator cells were small and potentially identifiable.

Group	Variable	Category	n (%) or summary
Music educators	Gender	Women	38 (45.8)
		Men	45 (54.2)
	Age	Valid n; mean ± SD; range	83; 45.49 ± 10.18; 19–65
	Educational status	Bachelor's	7 (8.4)
		Master's	19 (22.9)
		Doctorate	54 (65.1)
		Proficiency in art	3 (3.6)
	Years of instrumental experience	0–5	2 (2.4)
		6–10	1 (1.2)
		11–15	5 (6.0)
		≥16	75 (90.4)
	Weekly practice	1–2 days	18 (21.7)
		3–4 days	15 (18.1)
		5–6 days	28 (33.7)
		Every day	22 (26.5)
	Daily practice (valid n = 82)	Half an hour	13 (15.9)
		1–2 h	54 (65.9)
		3–4 h	11 (13.4)
		≥5 h	4 (4.9)
	Years teaching an instrument	0–5	7 (8.4)
		6–10	6 (7.2)
		11–20	18 (21.7)
		≥21	52 (62.7)
	Regular physical exercise	Yes	44 (53.0)
		No	39 (47.0)
	Instrument-related physical problem	Yes	41 (49.4)
		No	42 (50.6)
	Professionally diagnosed condition	Yes	17 (20.5)
		No	66 (79.5)
	Institutional distribution	15 coded institutions	Range: 1–14 educators per institution
Pre-service music teachers	Gender (valid n = 348)	Women	215 (61.8)
		Men	133 (38.2)
		Missing	1 (0.3 of total)
	Age	Valid n; mean ± SD; range	339; 22.54 ± 4.80; 18–48
	Year of study	1st	96 (27.5)
		2nd	106 (30.4)
		3rd	56 (16.0)
		4th	91 (26.1)
	Years of instrumental experience	0–5	194 (55.6)
		6–10	104 (29.8)
		11–15	40 (11.5)
		≥16	11 (3.2)
	Weekly practice	1–2 days	72 (20.6)

Group	Variable	Category	n (%) or summary
		2–3 days	3 (0.9)
		3–4 days	134 (38.4)
		5–6 days	89 (25.5)
		Every day	51 (14.6)
	Daily practice	Half an hour	55 (15.8)
		1–2 h	214 (61.3)
		3–4 h	69 (19.8)
		≥5 h	11 (3.2)
	Regular physical exercise	Yes	97 (27.8)
		No	252 (72.2)
	Instrument-related physical problem	Yes	185 (53.0)
		No	164 (47.0)
	Professionally diagnosed condition	Yes	29 (8.3)
		No	320 (91.7)
	Institutional distribution	15 coded institutions	Range: 7–64 pre-service teachers per institution

Table S1B. Principal Instrument/Voice Specializations

Group	Instrument/voice	n	%
Music educators	Piano	19	22.9
	Violin	19	22.9
	Cello	8	9.6
	Voice	7	8.4
	Flute	6	7.2
	Guitar	6	7.2
	Bağlama	5	6.0
	Viola	4	4.8
	Oud	3	3.6
	Combined/other	6	7.2
Pre-service music teachers	Violin	82	23.5
	Piano	52	14.9
	Guitar	46	13.2
	Flute	38	10.9
	Bağlama	33	9.5
	Cello	27	7.7
	Viola	26	7.4
	Voice	25	7.2
	Clarinet	10	2.9
	Other	10	2.9

Table S2. Questionnaire Structure, Scoring, and Representative Items

Block	Item no.	Content/short label	Response scoring	Analytic use
Educator contextual block	1	Post-practice pain/numbness/fatigue	1–5	Item-level only
Educator contextual block	2	Rest during practice	1–5	Item-level only
Educator contextual block	3	Knowledge of muscles and tendons	1–5	Item-level only
Educator contextual block	4	Effect of physical problems on practice	1–5	Item-level only
Educator contextual block	5	Consultation with a health professional	1–5	Item-level only
Educator contextual block	6	Student feedback about physical complaints	1–5	Item-level only
Educator contextual block	7	Information given to students about physical problems	1–5	Item-level only

Block	Item no.	Content/short label	Response scoring	Analytic use
Educator contextual block	8	Reminders before/during/after practice	1–5	Item-level only
Educator contextual block	9	Posture guidance	1–5	Item-level only
Educator contextual block	10	Information about physical structures at risk	1–5	Item-level only
Pre-service contextual block	1	Knowledge of muscles and tendons	1–5	Item-level only
Pre-service contextual block	2	Rest during practice	1–5	Item-level only
Pre-service contextual block	3	Effect of physical problems on practice	1–5	Item-level only
Pre-service contextual block	4	Information received about physical complaints	1–5	Item-level only
Pre-service contextual block	5	Frequency of physical complaints	1–5	Item-level only
Pre-service contextual block	6	Post-practice physical complaints	1–5	Item-level only
Pre-service contextual block	7	Sharing complaints with instrument educator	1–5	Item-level only
Pre-service contextual block	8	Information received about potential physical problems	1–5	Item-level only
Pre-service contextual block	9	Specialist-designed warm-up/relaxation guidance	1–5	Item-level only
Pre-service contextual block	10	Posture guidance	1–5	Item-level only
Shared disorder-knowledge block	11	Overuse syndrome	1 = none to 5 = completely	Summed; total 15–75
Shared disorder-knowledge block	12	Tendinitis	1 = none to 5 = completely	Summed; total 15–75
Shared disorder-knowledge block	13	Focal dystonia	1 = none to 5 = completely	Summed; total 15–75
Shared disorder-knowledge block	14	Carpal tunnel syndrome	1 = none to 5 = completely	Summed; total 15–75
Shared disorder-knowledge block	15	Thoracic outlet syndrome	1 = none to 5 = completely	Summed; total 15–75
Shared disorder-knowledge block	16	Cubital tunnel syndrome	1 = none to 5 = completely	Summed; total 15–75
Shared disorder-knowledge block	17	Trigger finger	1 = none to 5 = completely	Summed; total 15–75
Shared disorder-knowledge block	18	Entrapment neuropathies	1 = none to 5 = completely	Summed; total 15–75
Shared disorder-knowledge block	19	Disc herniation	1 = none to 5 = completely	Summed; total 15–75
Shared disorder-knowledge block	20	Temporomandibular disorder	1 = none to 5 = completely	Summed; total 15–75
Shared disorder-knowledge block	21	Dental alignment problems	1 = none to 5 = completely	Summed; total 15–75
Shared disorder-knowledge block	22	Task-specific tremor	1 = none to 5 = completely	Summed; total 15–75
Shared disorder-knowledge block	23	Fiddler's neck	1 = none to 5 = completely	Summed; total 15–75
Shared disorder-knowledge block	24	Scoliosis	1 = none to 5 = completely	Summed; total 15–75
Shared disorder-knowledge block	25	Kyphosis	1 = none to 5 = completely	Summed; total 15–75

Representative item wording: “How much knowledge do you have about overuse syndrome associated with instrumental performance?” and “How much knowledge do you have about focal dystonia associated with instrumental performance?” The complete copyrighted questionnaires are not reproduced; the table provides the structure and scoring required to interpret the analyses.

Table S3. Item-Level Psychometric Results for the 15-Item Knowledge Score

Item	Educator loading	Educator corrected item–total r	Educator α if deleted	Pre-service loading	Pre-service corrected item–total r	Pre-service α if deleted
Overuse syndrome	0.751	0.725	0.961	0.651	0.637	0.944
Tendinitis	0.787	0.779	0.960	0.763	0.728	0.942
Focal dystonia	0.814	0.801	0.959	0.813	0.775	0.941
Carpal tunnel syndrome	0.845	0.841	0.958	0.788	0.758	0.941
Thoracic outlet syndrome	0.889	0.858	0.958	0.840	0.795	0.941
Cubital tunnel syndrome	0.878	0.848	0.958	0.803	0.762	0.941
Trigger finger	0.830	0.797	0.959	0.842	0.801	0.941
Entrapment neuropathies	0.862	0.846	0.958	0.835	0.818	0.940
Disc herniation	0.775	0.747	0.960	0.783	0.781	0.941
Temporomandibular disorder	0.879	0.876	0.958	0.792	0.771	0.941
Dental alignment problems	0.748	0.739	0.960	0.668	0.652	0.944
Task-specific tremor	0.713	0.715	0.961	0.704	0.677	0.944
Fiddler's neck	0.594	0.605	0.963	0.625	0.627	0.945
Scoliosis	0.758	0.783	0.960	0.574	0.601	0.946
Kyphosis	0.701	0.722	0.961	0.605	0.625	0.945

Overall educator score: $n = 79$, $\alpha = 0.962$ (95% CI 0.944–0.974), $\omega = 0.963$, KMO = 0.913, Bartlett $\chi^2(105) = 1201.55$, $p < 0.001$. Overall pre-service score: $n = 341$, $\alpha = 0.946$ (95% CI 0.933–0.956), $\omega = 0.949$, KMO = 0.939, Bartlett $\chi^2(105) = 3929.77$, $p < 0.001$. Parallel analysis supported one factor in each group; extraction was maximum likelihood.

Table S4. Complete Body-Map Comparison Results

Anatomical region	Educators, n (%)	Pre-service, n (%)	OR (educator/pre-service)	95% CI	Fisher p	BH-FDR q
Right head/face	3 (3.6)	25 (7.2)	0.486	0.143–1.650	.324	.874
Left head/face	3 (3.6)	25 (7.2)	0.486	0.143–1.650	.324	.874
Neck	28 (33.7)	136 (39.0)	0.797	0.482–1.319	.450	.874
Right shoulder/posterior shoulder	21 (25.3)	93 (26.6)	0.932	0.539–1.614	.890	1.000
Left shoulder/posterior shoulder	23 (27.7)	105 (30.1)	0.891	0.523–1.517	.789	1.000
Right upper arm	3 (3.6)	48 (13.8)	0.235	0.071–0.775	.008	.056
Left upper arm	7 (8.4)	39 (11.2)	0.732	0.315–1.701	.556	.874
Right forearm	9 (10.8)	52 (14.9)	0.695	0.327–1.474	.386	.874
Left forearm	12 (14.5)	62 (17.8)	0.782	0.400–1.530	.521	.874
Right hand/wrist	11 (13.3)	128 (36.7)	0.264	0.135–0.516	<0.001	<0.001
Left hand/wrist	15 (18.1)	135 (38.7)	0.350	0.192–0.637	<0.001	.003
Right chest/back	2 (2.4)	15 (4.3)	0.550	0.123–2.452	.546	.874
Left chest/back	3 (3.6)	14 (4.0)	0.897	0.252–3.197	1.000	1.000
Right abdomen/lower back	4 (4.8)	37 (10.6)	0.427	0.148–1.233	.143	.630
Left abdomen/lower back	4 (4.8)	36 (10.3)	0.440	0.152–1.273	.143	.630
Genital region	0 (0.0)	1 (0.3)	0.000	0.056–34.458	1.000	1.000
Right upper leg	1 (1.2)	10 (2.9)	0.413	0.052–3.275	.699	.992

**Anatomical region**	**Educators, n (%)**	**Pre-service, n (%)**	**OR (educator/pre-service)**	**95% CI**	**Fisher p**	**BH-FDR q**
Left upper leg	0 (0.0)	10 (2.9)	0.000	0.011–3.338	.220	.808
Right lower leg	3 (3.6)	10 (2.9)	1.271	0.342–4.726	.722	.992
Left lower leg	2 (2.4)	8 (2.3)	1.052	0.219–5.050	1.000	1.000
Right foot/ankle	3 (3.6)	8 (2.3)	1.598	0.415–6.160	.449	.874
Left foot/ankle	2 (2.4)	12 (3.4)	0.693	0.152–3.159	1.000	1.000
